# Adaptive designs undertaken in clinical research: a review of registered clinical trials

**DOI:** 10.1186/s13063-016-1273-9

**Published:** 2016-03-19

**Authors:** Isabella Hatfield, Annabel Allison, Laura Flight, Steven A. Julious, Munyaradzi Dimairo

**Affiliations:** School of Mathematics & Statistics, Newcastle University, Herschel Building, Newcastle upon Tyne, NE1 7RU UK; ScHARR, University of Sheffield, 30 Regent Street, Sheffield, S1 4DA UK; MRC Biostatistics Unit, Cambridge Institute of Public Health, Forvie Site, Robinson Way, Cambridge Biomedical Campus, Cambridge, CB2 0SR UK

**Keywords:** Adaptive design, Clinical trial, Flexible design

## Abstract

Adaptive designs have the potential to improve efficiency in the evaluation of new medical treatments in comparison to traditional fixed sample size designs. However, they are still not widely used in practice in clinical research. Little research has been conducted to investigate what adaptive designs are being undertaken. This review highlights the current state of registered adaptive designs and their characteristics. The review looked at phase II, II/III and III trials registered on ClinicalTrials.gov from 29 February 2000 to 1 June 2014, supplemented with trials from the National Institute for Health Research register and known adaptive trials. A range of adaptive design search terms were applied to the trials extracted from each database. Characteristics of the adaptive designs were then recorded including funder, therapeutic area and type of adaptation. The results in the paper suggest that the use of adaptive designs has increased. They seem to be most often used in phase II trials and in oncology. In phase III trials, the most popular form of adaptation is the group sequential design. The review failed to capture all trials with adaptive designs, which suggests that the reporting of adaptive designs, such as in clinical trials registers, needs much improving. We recommend that clinical trial registers should contain sections dedicated to the type and scope of the adaptation and that the term ‘adaptive design’ should be included in the trial title or at least in the brief summary or design sections.

**Electronic supplementary material:**

The online version of this article (doi:10.1186/s13063-016-1273-9) contains supplementary material, which is available to authorized users.

## Review

### Background

Adaptive designs (ADs) have the potential to improve efficiency in the evaluation of new medical treatments in practice and alleviate some of the shortcomings of fixed sample size designed trials when used appropriately [1]. However, ADs are not widely used routinely in clinical trial research despite the prominence given to them in the statistical literature [2–5]. Initiatives, predominately from a pharmaceutical drug development perspective, have been undertaken to understand and address some of the perceived barriers to the uptake of ADs in routine practice when they are considered appropriate [5–9]. Most importantly in this sector, regulatory bodies have drafted guidance documents or reflection papers on ADs to facilitate their use [6, 10–12].

Whilst it would be logical to infer that these initiatives would lead to an increase in the application of ADs, little research has been done to investigate if this is the case [2, 8, 13, 14]. Advocates for ADs suggest their use is increasing while opponents say otherwise [1, 15].

Recent studies have highlighted barriers to the use of ADs including: a lack of practical knowledge and experience, insufficient access to case studies of ADs, lack of awareness of types of AD, unfamiliarity with ADs and fear of jeopardising chances of regulatory approval [4, 5, 16, 17].

The perceived barriers led to the motivation for this study, which aims to review the types of registered ADs in use and explore in more detail their characteristics. The objective of the review in this paper is to highlight the current state of ADs and raise awareness regarding the type of ADs being implemented in clinical trial research. The specific objectives of this review are to explore:

1. The number of trials designed and conducted as ADs
2. The type of ADs being implemented with particular emphasis on confirmatory trials
3. The most common therapeutic areas where certain types of ADs are being used
4. The distribution of ADs by geographic location, trial phase and funder or sponsor
5. The trial characteristics of ADs
6. Trends in the use of ADs by trial phase and funder
7. The adequacy of ClinicalTrials.gov [18] in capturing AD trials

### Methods

#### Literature search

The World Health Organization (WHO) register [19] was used to carry out a feasibility study. The results informed the choice of databases for the main review, which involved searching the ClinicalTrials.gov database [18] and the National Institute for Health Research (NIHR) database [20] for AD trials, subject to pre-specified inclusion criteria. The search was restricted to dates between 29 February 2000 (when ClinicalTrials.gov [18] became available to the public) and 1 June 2014.

#### Feasibility

A comprehensive feasibility study was conducted using the WHO register [19]. Trials registered on 25 June from the years 2009 to 2013 inclusive were chosen for this exercise. The date, 25 June, was randomly selected using R Studio and the years restricted to 2009 to 2013 because time constraints required us to limit the number of trials reviewed and we anticipated the number of ADs being larger in more recent years. For trials satisfying the main study inclusion criteria (details presented in the Eligibility criteria section), two reviewers (AA and LF) independently and manually ascertained whether they could be classified as adaptive or not. Decisions were aided by any available material related to the study such as protocols and publications.

**Fig. 1:**
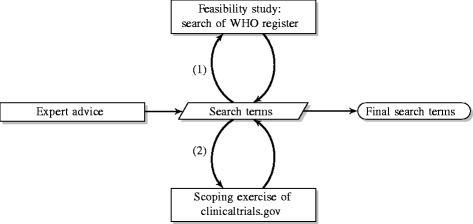
Search strategy. A flow diagram of the decision-making process used to determine the search terms. *WHO* World Health Organization

A list of AD related search terms was applied to all trials meeting the inclusion criteria to check whether the adaptive trials found manually were also identified using the search terms. Due to the restrictive nature of the searching algorithm of the WHO register (limited to lay and scientific titles), there was poor agreement between the two approaches. Thus, any trials that did not highlight the adaptive nature of the study in these titles would not be identified by the search. The search terms were updated based on these findings as illustrated in Fig. 1. In addition, trial phase could not be ascertained for a large proportion of trials and the adaptive nature of the trials was not often described in detail or missing, implying a major limitation of using the WHO register. Hence, the main review was restricted to ClinicalTrials.gov [18], as it has better flexibility and is improved in filtering records, data completeness and searching options.

#### Search strategy

A list of AD related search terms was compiled (see Additional file 1). This was an iterative process (see Fig. 1) with the chosen terms based on results from the feasibility study, the opinions of experts in the field of ADs [21] and a scoping exercise using ClinicalTrials.gov [18] to eliminate redundant terms. The search terms were applied to trials meeting the inclusion criteria using the Boolean OR operator. These were then extracted and one reviewer (IH) confirmed whether the trials were truly adaptive in design in consultation with other researchers (LF and MD) when necessary, as a form of quality control.

#### Data sources

The main source for the review was ClinicalTrials.gov [18], as it is a large database and includes unpublished trials. We decided to use ClinicalTrials.gov [18], as opposed to peer-reviewed publications, as it has the potential for real-time data capture, thus reducing the time lag between trial commencement and publication, which can take a number of years. It also has the potential to reduce the publication bias found in peer-reviewed journals – positive findings of successful trials are more likely to be published than those with negative findings [22]. This could potentially downplay the number of ADs as one of their main features is the ability to stop trials for futility, i.e. if the results are negative. In contrast, registration of all trials is now mandatory, so using ClinicalTrials.gov [18] will obviate this problem provided the information given is complete. The database does have its own limitations, however, and so the search from ClinicalTrials.gov [18] was supplemented with trials identified from the NIHR register [20], which contains more information, and known adaptive trials from contacts with trialists within the pharmaceutical and public sector.

For the latter, contacts were made through personalised emails, specialised group emails (such as MedStats, Google group and the UK CTU infrastructure network of senior statisticians), and specialised group posts (such as LinkedIn targeting Statistics in the Pharmaceutical Industry and ADs working groups – 1601 members as of 10 July 2014). Originally, it was intended to include Medical Research Council (MRC) funded trials as supplementary material. However, it was not possible to find an up-to-date list of trials for this funding body and so these could not be included in the review.

Trials from the two supplementary sources were linked back to ClinicalTrials.gov [18] to extract additional information of interest. Duplicates were checked using the unique trial registration number and removed before analysis. The data source was left-truncated at 29 February 2000 and right-truncated on 1 June 2014.

#### Dealing with missing data

Chief investigators were contacted with a request to respond within 4 weeks to reduce missing data. If the missing information was needed to determine whether or not the trial design was adaptive, the trial was excluded from the review. If the trial was known to be adaptive but some other information was missing (e.g. sample size), the trial was included in the review but missing data highlighted.

#### Eligibility criteria

Clinical trials were eligible to be included if the following criteria were satisfied:

- The trial investigates an intervention(s) on humans with a comparator.
- It is phase II, III or II/III.
- It was left-truncated on 29 February 2000 and right-truncated on 1 June 2014.
- Trial documents are written in English.

#### Quality control

A second reviewer (MD) validated all phase III ADs and two reviewers (LF and MD) validated any other trials where clarification was required.

To assess the adequacy of ClinicalTrials.gov [18] in capturing AD trials, a search of published trials using MEDLINE was performed. With MEDLINE, it is possible to search the abstracts and titles of published trials more comprehensively than with ClinicalTrials.gov [18]. The anticipation is that more trials would be found through this route. Its main limitation is that it does not have ongoing trials unless the trial has published the protocol.

The filters ‘English’, ‘humans’, ‘2000 to current’, ‘clinical trial all’, ‘controlled clinical trial’, ‘pragmatic clinical trial’, ‘randomised controlled trial’ (RCT) and ‘full-text’ were applied, giving 2079 trials (as of 1 June 2014). A random sample of 300 trials was selected and the design and phase of the trials extracted by three reviewers (MD, AA and LF). The registration of any AD trials on ClinicalTrials.gov [18] was checked and a search of those trials included in the review undertaken to ascertain the number and percentage of ADs missed and picked up by the search.

#### Data collection

The following information was collected from the included trials and recorded on an Excel spreadsheet:

- Whether the trial was truly adaptive and the nature of the adaptation if so
- The stopping rule, for example, futility or efficacy
- The year of registration and completion
- The nature and duration of the primary outcome
- The expected total sample size
- The scope of the study (national or international)
- The country of the lead chief investigator
- The nature of the experimental intervention and the comparator and the number of treatment arms
- The funder or sponsor of the study
- The current state of the trial, for example, terminated, ongoing or completed
- The therapeutic area under study
- The population under study
- Whether or not the trial is published
- Reason for termination on those trials that terminated early

For phase III trials, additional information was also collected:

- Other design characteristics, for example, parallel group
- Nature of the primary hypothesis of interest, for example, superiority

#### Main outcome measures

The main outcome measures were:

1. The types of ADs
2. The frequency of ADs

#### Outline of analyses

We used descriptive summary statistics depending on the nature of the variables and graphs for presentation. Results were also stratified by phase and funder. The number of ADs per 10,000 registered trials by time period and 95 % confidence intervals (CIs) were produced and graphed to explore the trend in the use of ADs.

### Results

#### Study selection

**Fig. 2:**
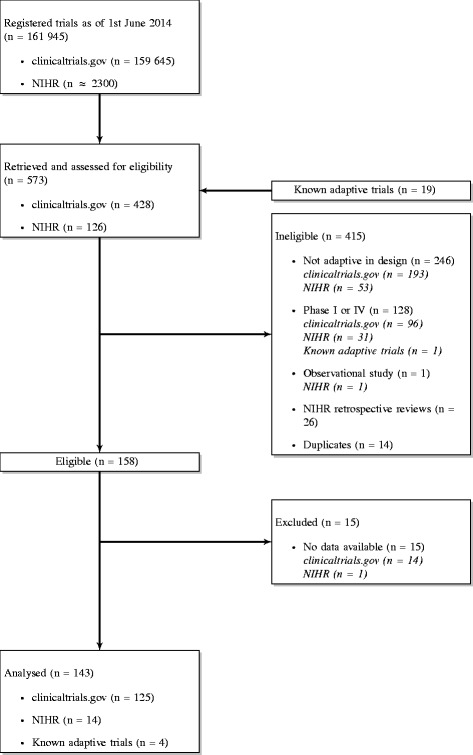
Screening process. A flow diagram showing the review process including reasons for exclusion of trials. *NIHR* National Institute for Health Research

As of 1 June 2014, 159,645 trials were registered on ClinicalTrials.gov [18] and approximately 2300 on the NIHR register [20]. Of these, 554 were assessed for eligibility together with 19 known adaptive trials. Only 158 were eligible for further review and analysis. Among the reasons for ineligibility were: not adaptive in design (*n*=246), phase I or IV (*n*=128), observational study (*n*=1), NIHR retrospective reviews (*n*=26) and duplicates (i.e. known trials that were already captured in the search) (*n*=14). A further 15 trials were excluded from the analysis because information required to determine the trial design was missing, leaving a total of 143 trials for the analysis. Figure 2 shows a flow diagram of the screening process. Table 1 describes a sample of trials included in the review.

**Table 1:** Brief descriptions of a sample of identified confirmatory ADs captured in the review

Trial registration number	Description
NCTO1230775	This is a private-sector-funded two-stage confirmatory AD with sample size re-estimation (SSR) at the first interim analysis applying the methodology proposed by Bauer and Kohne [25] using *P* value combination procedures. It is a double-blinded RCT investigating the efficacy and safety of a drug Anagrelide Retard in patients with essential thrombocythaemia at certain defined risk criteria.
NCT01555710	The MATISSE study is a private-sector-sponsored open-label RCT with an active comparator, adaptive group sequential design (GSD) with SSR at the interim analysis evaluating the efficacy of palifosfamide-tris, in combination with carboplatin and etoposide chemotherapy in chemotherapy naive patients with extensive-stage small-cell lung cancer.
NCT00268476	The STAMPEDE study is a phase II/III RCT with a multi-arm multi-stage AD investigating five treatments in combination with hormone therapy for patients with locally advanced or metastatic prostate cancer with options to drop futile arms or add new investigation arms during the trial. The trial is predominantly funded by the UK public sector. Sydes et al. [26, 27] describe the rationale and design aspects of the trial. James et al. [28] present the first interim results with decisions to discontinue certain intervention arms.
NCT01545232	The PROPPR study is a GSD with SSR, funded by the public sector in the USA, investigating the effectiveness and safety of transfusing patients with severe trauma and major bleeding using plasma, platelets and red blood cells in a 1:1:1 ratio compared with a 1:1:2 ratio. Baraniuk et al. [29] provide the detailed rationale and design of the trial. The two co-primary outcomes were separately monitored using a two-sided O’Brien and Fleming [30] boundary with Lan and DeMets [31] alpha spending function based on numbers of events for each of the two comparisons. SSR was performed prior to the first efficacy interim analysis. Holcomb et al. [32] report the trial results in the *Journal of the American Medical Association*.
NCT01336530	The PREVAIL study is a private-sector-funded randomised parallel-group double-blind placebo-controlled therapeutic confirmatory multicentre trial with four intervention arms, inclusive of the comparator. The trial is a Bayesian adaptive GSD with two interim analyses, possible SSR after the first or second interim analysis and drop-the-loser approach (option to drop futile intervention arms). Holmes et al. [33] report the results of the trial.
NCT00497146	The PRIMO study is a private-sector-funded trial evaluating the effects of a drug (paricalcitol) on cardiac structure and function over 48 weeks in patients with stage 3/4 chronic kidney disease who had left ventricular hypertrophy. The trial is an information-based GSD with SSR. Pritchett et al. [34] provide the design details and rationale, and Thadhani et al. [35] present the findings.
ISRCTN 06473203	The STAR study is a multi-stage operational seamless II/III RCT publicly funded by the NIHR Health Technology Assessment. The trial investigates the effect of a novel drug-free-interval strategy compared to the standard treatment strategy in the first-line treatment of advanced renal cell carcinoma [36].
ISRCTN90061564	FOCUS4 is a MAMS seamless II/III design investigating multiple treatments in multiple population enriched biomarkers in oncology. Kaplan et al. [37] provide a detailed description of the design, its rationale, statistical properties and implementation tools.

#### Study characteristics

**Fig. 3:**
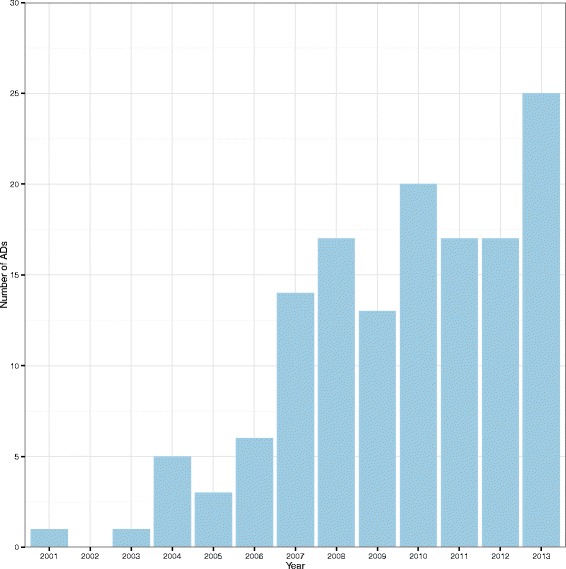
Bar chart showing the number of ADs per year. Only complete years are represented. *AD* adaptive design

**Fig. 4:**
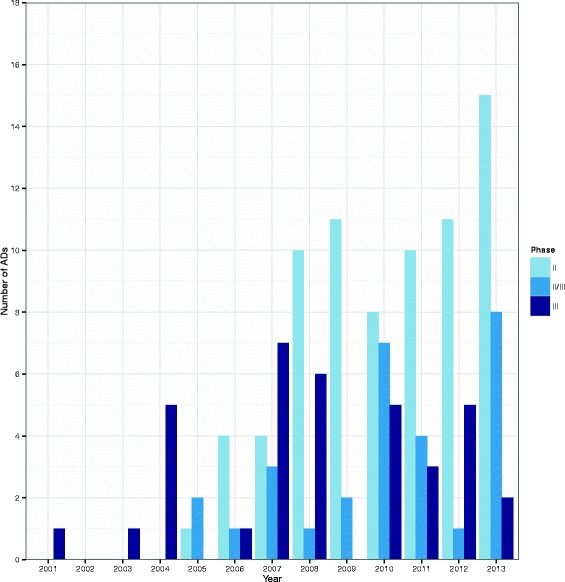
Bar chart showing the number of ADs per year by phase. Only complete years are represented. *AD* adaptive design

**Fig. 5:**
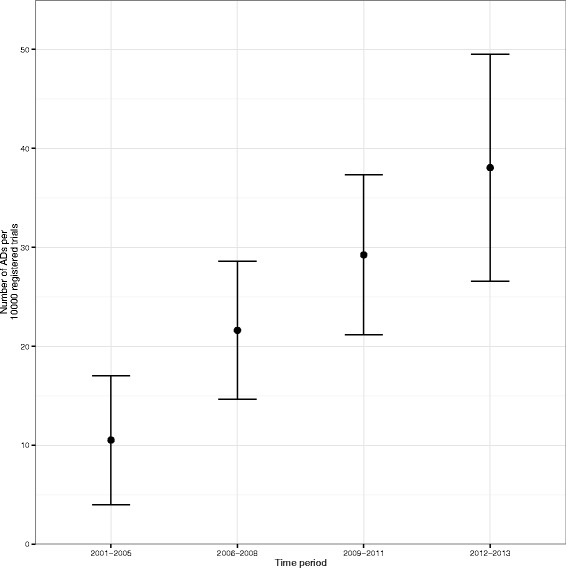
Forest plot of the number of ADs per 10,000 registered trials by each time period. Only complete years are represented. *AD* adaptive design

**Frequency and type of ADs** Figure 3 provides a bar chart of the number of ADs per year whilst Fig. 4 provides a clustered bar chart of the number of ADs per year by phase. Table 2 shows the number of ADs per 10,000 registered trials by time period – years were grouped together due to the small number of ADs – together with a 95 % CI. This information is also represented on a forest plot in Fig. 5. On the face of it, it appears that the use of ADs has increased over time. However, as it has not been possible to record all ADs, these results should be taken with caution.

**Table 2:** Number of ADs (95 % CI) per 10,000 registered trials per time period

Year	Total number	Total number of	Number of ADs per 10,000
	of ADs	registered trials	registered trials (95 % CI)
2001–2005	10	9502	11 (4, 17)
2006–2008	37	17,116	22 (15, 29)
2009–2011	50	17,097	29 (21, 37)
2012–2013 ^a^	46	11,037	38 (27, 50)

*AD* adaptive design, *CI* confidence interval

^a^Results from 2014 excluded as this was only a partial year

**Fig. 6:**
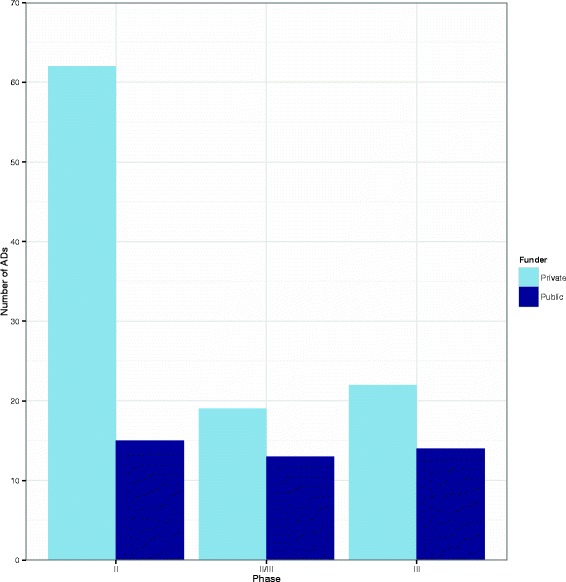
Clustered bar chart showing the number of ADs by phase and funder. *AD* adaptive design

Figure 6 shows a clustered bar chart of the frequency of ADs by phase and funder. This suggests that ADs are most commonly used in privately funded phase II trials. The ratio of private to publicly funded trials appear to be similar in phases II/III and III.

The type of adaptation undertaken varies according to phase for both types of funder:

- For phase II trials, GSD and dose selection (DS) designs are the most common types of adaptation.
- For phase II/III trials, GSD/seamless and DS/seamless are the most common types of adaptation.
- In phase III trials, GSD is the most common type of adaptation (Table 3).

**Table 3:** Type of adaptation stratified by phase and funder

Type of adaptation	Phase II		Phase II/III		Phase III		All phases
	Private	Public	Private	Public	Private	Public	Total
GSD only	10	6	0	2	10	10	38
SSR only	1	0	0	0	4	2	7
DS	25	3	0	0	0	0	28
Dose escalation (DE)	3	2	0	0	1	0	6
Seamless	0	0	3	1	0	0	4
GSD and SSR	1	0	0	0	2	0	3
GSD and DS	13	2	0	1	4	2	22
GSD and DE	1	1	0	0	0	0	2
GSD and seamless	0	0	3	6	0	0	9
SSR and DS	4	1	0	0	0	0	5
Seamless and SSR	0	0	3	2	0	0	5
Seamless and DS	1	0	10	1	0	0	12
Interim analysis	1	0	0	0	0	0	1
Interim analysis and SSR	0	0	0	0	1	0	1

*DE* dose escalation, *DS* dose selection, *GSD* group sequential design, *SSR* sample size re-estimation

**Fig. 7:**
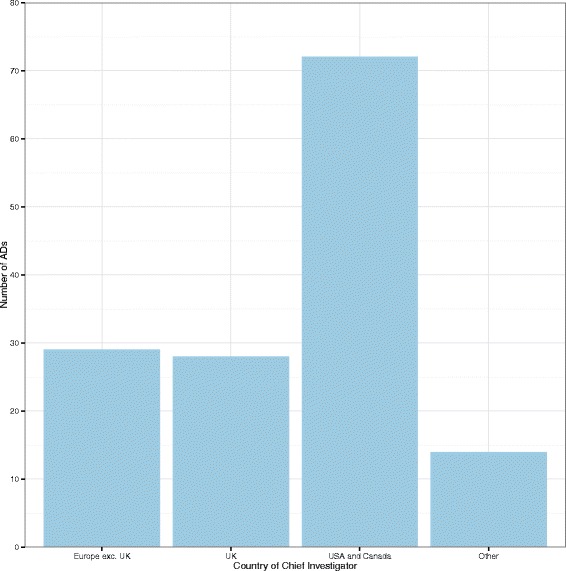
Bar chart showing the number of ADs by geographic location. *AD* adaptive design

**Geographic location** Figure 7 shows a bar chart of the number of ADs by geographical location. The majority of ADs were carried out in the US and Canada, whilst the number carried out in the UK was similar to the number carried out in the rest of Europe.

**Other study characteristics** Across all phases and funders, ADs are most commonly used in oncology trials. However, they can be used in a wide range of therapeutic areas (see Additional file 2). For both sectors, the median sample size was larger for phase III trials as expected and the majority of trials investigated one comparator arm though there were several multi-arm trials (see Additional file 2).

The main reason for early termination in those trials that terminated after enrolment was futility (Table 4). The additional data collected for phase III trials showed that they were all superiority trials and the majority were parallel in design (one factorial design).

**Table 4:** Reasons for early termination of a trial

Reason	Phase II		Phase II/III		Phase III	
	Private	Public	Private	Public	Private	Public
Poor recruitment	1	0	1	0	0	1
Efficacy	0	0	0	0	1	0
Futility	3	2	3	0	4	1
Safety	0	0	0	0	0	1
Financial	1	0	1	0	0	0
Other	0	0	0	0	1	0
All ^a^	5	2	5	0	6	3

^a^Only trials terminated after enrolment included

Some characteristics of the trials depend on the source of funding:

- For private funders, the most common type of primary outcome is continuous across all phases, whilst for publicly funded trials the outcome is phase dependent: continuous being the most common in phase II trials and binary in phases II/III and III.
- The most common stopping rule is efficacy/safety for privately funded trials across all phases. In comparison, for publicly funded trials, the most common stopping rule differs across phases: efficacy at phase II and efficacy/safety/futility at phases II/III and III.
- Privately funded trials are commonly international studies whilst publicly funded trials are most commonly national.
- The median duration of the primary outcome is greatest for phase II/III publicly funded trials and phase III privately funded trials.

Of the 76 trials that were either completed or terminated after recruitment (as of September 2014), 43 (56 %) had published their results (as of May 2015). Of these, 27 (63 %) had either published the results within 2 years of study completion, or published the interim analysis results before trial completion.

#### Quality control and efficiency of ClinicalTrials.gov in capturing ADs

**Fig. 8:**
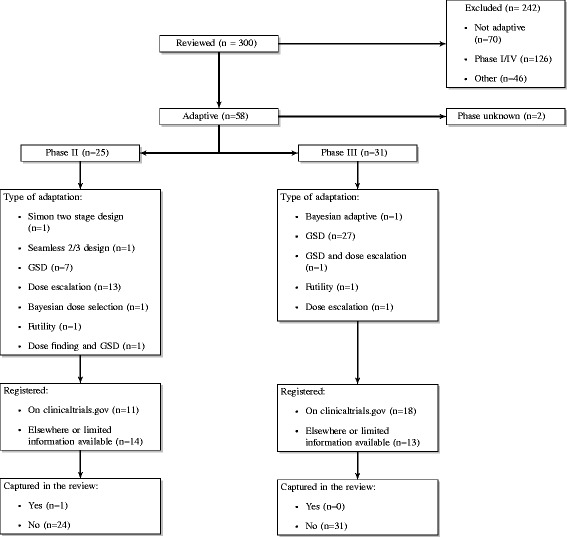
MEDLINE process. A flow diagram of the MEDLINE search process. *GSD* group sequential design

The search of MEDLINE suggests that a number of AD trials on ClinicalTrials.gov [18] were missed by the search strategy. Of the 300 randomly selected trials from MEDLINE, 29 (10 %) satisfied the inclusion criteria, were adaptive in design and were registered on ClinicalTrials.gov [18]. Only one of these (3 %) was captured in the review. The remaining 28 (97 %) were either registered elsewhere or there was limited information as to whether or not the trial was registered. Figure 8 shows the screening process for the MEDLINE search and includes details on the types of AD found.

### Discussion

#### Main findings

The results suggest that uptake of ADs is now gaining traction and increasing. The most popular type of AD in phase III trials is the GSD. This is most likely because it is well established in the statistical literature and it is described by regulators as being well understood [12]. Trialists may, therefore, be more inclined to use designs that they know well.

Oncology appears to be the main therapeutic area where ADs are undertaken. This could be for a number of reasons. Oncology is an area where regulators and the research community may be receptive to adaptation and may be more willing to accept such a design. If there are limitations in current standard care for a type of cancer, the research community may need to know quickly if a new treatment is promising so patients can have access to it and cross over from standard care in the trial. Following on from the previous point, it may take a number of years to get a definitive answer based on survival and so the research community may be willing to make decisions on treatment based on interim results on endpoints such as disease-free survival until the definitive results come in.

Whilst oncology is the main therapeutic area in which ADs are conducted, there is diversity in the therapeutic area in which ADs can be undertaken (see Additional file 2). The underutilisation in some areas may be due to limited examples of the designs being applied, which both forms a deterrent and vicious circle. Counter to this, in oncology, there may be a virtuous circle of trialists having a number of case studies to which they can refer and so they can see practically how they can be implemented.

The majority of ADs are phase II trials, which reflects the literature and regulatory guidance regarding the wide scope of adaptation in early-phase trials due to the exploratory nature of the objectives [8, 13, 14]. Whilst phase II/III and phase III trials were evenly spread across funders, there was a much higher proportion of privately funded phase II trials than publicly funded. This is mainly due to the desire by the private sector to reduce drug development time and costs [8]. The private sector may also undertake more early-phase trials, as they often investigate unlicensed drug interventions, whereas the interventions studied by the public sector are more varied: licensed and unlicensed treatments as well as health technologies.

Another important finding is the reason for early termination of a trial, with futility being the main reason. Fewer trials stopped early for efficacy suggesting there is a reluctance amongst the involved parties (funders, trialists, and data monitoring and ethics committees) to stop early. This possibly could be due to concerns about the robustness of AD methods when stopping for efficacy. On the other hand, they may be willing to stop early for futility as it is good for both ethical and financial reasons. Also, the consequences of stopping early for futility could be perceived as less pronounced. It could mean a new treatment is no more effective than usual care so usual care remains as the standard treatment.

#### Limitations

The main data source ClinicalTrials.gov [18] posed a few issues. Firstly, many of the search terms associated with adaptive methods were redundant in the register. We, therefore, may have missed some AD trials where the terminology associated with their methodology was not used. In addition, some trials were written retrospectively into the register and did not state whether interim analysis, futility assessment using conditional power, or SSR were planned and carried out, which may have caused us not to identify some ADs. The search of MEDLINE also suggests that the search strategy did not capture all ADs, possibly due to the limited information available on ClinicalTrials.gov [18]. The register does not include sections for trialists to provide further information regarding the nature and scope of any adaptation. For some of the terminated or completed trials, no contact details were available and so the data extraction could not be performed. Originally, it was intended to include MRC funded trials as supplementary material. However, it was not possible to find an up-to-date list of trials and so these could not be included in the review. The review highlights ADs that have been well reported and are readily available through ClinicalTrials.gov.

In regards to trial designs, it is not possible to differentiate between operational and inferential seamless designs, hence the reason seamless designs have been grouped into one category in the analysis. This may be due to confusion or a lack of knowledge of the difference between operational and inferential seamless designs. There were fewer SSRs and futility assessments through conditional power in the review than we expected, possibly because they are being misclassified or not viewed as an AD. It was also difficult to find the nature of the adaptation in phase II trials and to establish whether or not DS/dose escalation (DE) trials were truly adaptive in design.

Another limitation of the review is that the data sources used favour publicly funded trials. Whilst we could have extended our sources to include pharmaceutical company websites, we did not think it feasible [14] and it may bias results to companies with better websites.

Recently, an arm of the Food and Drug Administration reviewed submission protocols and found 136 phase II or III ADs [23], highlighting underestimation of our review though the ClinicalTrials.gov [18] register. However, one point of reassurance is that based on our review, the frequency of certain types of AD, such as GSDs and SSRs, is consistent with this regulator review.

Finally, chief investigators were only contacted once and given a deadline of 4 weeks to reply to reduce missing data. Email reminders could have been sent to reduce missing data further.

Given the limitations, we do not feel that the results are invalidated as the objectives were to investigate the types of AD trials being undertaken and in which therapeutic areas. We feel we have achieved this even though the number of trials is unreported.

#### Implications and recommendations

The proportion of completed trials that were published and could be used as case studies is low at only 56 %. Whilst this is not unique to ADs, it is critical to have case studies of such complex trials to provide trialists with the information needed to choose an appropriate AD and to demystify the fear that ADs are a no-go area. Though we have not managed to extract an exhaustive list of ADs, a list of those captured in the review is provided (see Additional file 3) for trialists to use as practical case studies. We recommend publishing after completion of a trial to expand the list of trials available as case studies.

The inability to capture all ADs on ClinicalTrials.gov [18] using the search terms has highlighted that it is suboptimal for the registration of ADs. Since it can take several years from a trial starting to publishing results, adequate reporting of ADs in clinical trial registers gives other trialists the opportunity to see how ADs are currently being used and perhaps alleviate some of the barriers associated with ADs (for example, that funders and regulators are against the use of ADs). One of the issues with ADs is that their point estimates and CIs based on traditional analysis for fixed designs are biased. An important part of clinical research is to undertake systematic reviews of the evidence and to do so it is important to know which studies are adaptive and which are not. We recommend that clinical trial registers should contain sections dedicated to the type of AD and scope of the adaptation, including stopping rules, if this is a feature of the design. We also suggest that the title of the trial, or the brief summary or design sections, should contain the words ‘adaptive design’ so that it can be easily retrieved in a search. A modification to the CONSORT statement could help with the improvement of the reporting of AD trials [24].

## Conclusions

The use of ADs appears to be increasing, though we have not been able to capture all ADs in the review. There may be disease areas in which ADs are being underutilised and types of AD not being implemented when they would be appropriate.

## Additional files

Additional file 1 Search terms. PDF with a table of the final selection of search terms used in the review. (PDF 6.19 kb)

Additional file 2 Table of summary statistics. PDF containing a table of summary statistics by phase and funder type. Counts and percentages are presented for categorical variables whilst medians and interquartile ranges are presented for continuous variables. (PDF 19.5 kb)

Additional file 3 Case studies. XLSX file containing a list of the trials used in the review. Trial number, title and URL are provided. (XLSX 35.2 kb)

## Abbreviations

AD: adaptive design
CI: confidence interval
DE: dose escalation
DS: dose selection
GSD: group sequential design
MRC: Medical Research Council
NIHR: National Institute for Health Research
RCT: randomised controlled trial
SSR: sample size re-estimation
WHO: World Health Organization
