# Supplementary material for: Adaptive designs undertaken in clinical research: a review of registered clinical trials
Source: Trials. 2016 Mar 19;17:150. doi: 10.1186/s13063-016-1273-9 (PMC4799596; doi:10.1186/s13063-016-1273-9)
Supplement: Additional file 1 — Search terms. PDF with a table of the final selection of search terms used in the review. (PDF 6.19 kb) [file 13063_2016_1273_MOESM1_ESM.pdf]

Search terms
Adaptive
Interim
Dose selection
Bayesian
Futility
Enrichment
Stopping rule
Seamless
Group sequential
Go/no go
Preplanned
MAMS/Multi-stage/multiple stage/multiple arm
Active learning
Accumulating data
Continuous reassessment
Reanalysis
Pick the winner
Internal pilot
Drop the loser
Dose escalation
Sample size adjustment/Sample size re-estimation/Sample size modification
