# Supplementary material for: Adaptive designs undertaken in clinical research: a review of registered clinical trials
Source: Trials. 2016 Mar 19;17:150. doi: 10.1186/s13063-016-1273-9 (PMC4799596; doi:10.1186/s13063-016-1273-9)
Supplement: Additional file 2 — Table of summary statistics. PDF containing a table of summary statistics by phase and funder type. Counts and percentages are presented for categorical variables whilst medians and interquartile ranges are presented for continuous variables. (PDF 19.5 kb) [file 13063_2016_1273_MOESM2_ESM.pdf]

Variable	Phase II		Phase II/III		Phase III		All Phases
	Private (n=60)	Public (n=15)	Private (n=19)	Public (n=13)	Private (n=22)	Public (n=14)	Total (n=143)
Disease area							
Oncology	10 (17)	4 (27)	4 (21)	6 (46)	7 (32)	4 (29)	35 (24)
Stroke	0 (0)	0 (0)	0 (0)	0 (0)	1 (5)	1 (7)	2 (1)
Mental health	8 (13)	1 (7)	3 (16)	1 (8)	1 (5)	1 (7)	15 (10)
Dementia	3 (5)	0 (0)	1 (5)	0 (0)	0 (0)	0 (0)	4 (3)
Cardiology	2 (3)	2 (13)	0 (0)	0 (0)	2 (9)	2 (14)	8 (6)
Musculoskeletal	9 (15)	1 (7)	3 (16)	1 (8)	1 (5)	0 (0)	15 (10)
Respiratory	3 (5)	0 (0)	3 (16)	0 (0)	1 (5)	1 (7)	8 (6)
Immunodeficiency	3 (5)	0 (0)	0 (0)	1 (8)	0 (0)	1 (7)	5 (3)
Primary care	3 (5)	2 (13)	0 (0)	0 (0)	0 (0)	1 (7)	6 (4)
Diabetes	2 (3)	0 (0)	1 (5)	0 (0)	3 (14)	0 (0)	6 (4)
Oral and gastroenterology	9 (15)	1 (7)	1 (5)	1 (8)	1 (5)	2 (14)	15 (10)
HIV	2 (3)	1 (7)	1 (5)	1 (8)	3 (14)	1 (7)	9 (6)
Learning difficulties	2 (3)	1 (7)	0 (0)	0 (0)	1 (5)	0 (0)	4 (3)
Other[1]	4 (7)	2 (13)	2 (11)	2 (15)	1 (5)	0 (0)	11 (8)
Stopping rules							
Efficacy	8 (13)	7 (47)	2 (11)	1 (8)	3 (14)	2 (14)	23 (16)
Safety	0 (0)	0 (0)	1 (5)	0 (0)	0 (0)	0 (0)	1 (1)
Futility	0 (0)	0 (0)	0 (0)	1 (8)	0 (0)	0 (0)	1 (1)
Efficacy/safety	43 (72)	5 (33)	12 (63)	4 (31)	13 (59)	4 (29)	81 (57)
Efficacy/futility	1 (2)	1 (7)	0 (0)	2 (15)	1 (5)	1 (7)	6 (4)
Safety/futility	0 (0)	0 (0)	0 (0)	0 (0)	0 (0)	1 (7)	1 (1)
Efficacy/safety/futility	8 (13)	2 (13)	4 (21)	5 (38)	5 (23)	6 (43)	30 (21)
National or international							
National	26 (43)	11 (73)	4 (21)	11 (85)	6 (27)	12 (86)	70 (49)
International	32 (53)	4 (27)	15 (79)	2 (15)	16 (73)	2 (14)	71 (50)

continued on next page

continued from previous page

Variable	Phase II		Phase II/III		Phase III		All Phases
	Private (n=60)	Public (n=15)	Private (n=19)	Public (n=13)	Private (n=22)	Public (n=14)	Total (n=143)
Unknown	2 (3)	0 (0)	0 (0)	0 (0)	0 (0)	0 (0)	2 (1)
State of study[2]							
Active, not recruiting	0 (0)	0 (0)	0 (0)	2 (15)	0 (0)	0 (0)	2 (1)
Recruiting	17 (28)	12 (80)	6 (32)	8 (62)	3 (14)	5 (36)	51 (36)
Ongoing after recruitment	6 (10)	0 (0)	1 (5)	0 (0)	4 (18)	1 (7)	12 (8)
Completed	30 (50)	1 (7)	7 (37)	3 (23)	9 (41)	5 (36)	55 (38)
Terminated after recruitment	5 (8)	2 (13)	5 (26)	0 (0)	6 (27)	3 (21)	21 (15)
Terminated before enrolment	2 (3)	0 (0)	0 (0)	0 (0)	0 (0)	0 (0)	2 (1)
Population							
< 16	3 (5)	2 (13)	3 (16)	1 (8)	1 (5)	0 (0)	10 (7)
≥ 16	52 (87)	11 (73)	12 (63)	9 (69)	19 (86)	13 (93)	116 (81)
> 50 only[3]	4 (7)	1 (7)	4 (21)	1 (8)	1 (5)	1 (7)	12 (8)
All ages	1 (2)	1 (7)	0 (0)	2 (15)	1 (5)	0 (0)	5 (3)
Primary outcome(s)							
Binary	8 (13)	3 (20)	0 (0)	4 (31)	5 (23)	4 (29)	24 (17)
Continuous	47 (78)	11 (73)	16 (84)	4 (31)	9 (41)	4 (29)	91 (64)
Time to event	1 (2)	0 (0)	2 (11)	3 (23)	6 (27)	3 (21)	15 (10)
Ordinal	2 (3)	1 (7)	0 (0)	2 (15)	0 (0)	1 (7)	6 (4)
Categorical	1 (2)	0 (0)	0 (0)	0 (0)	1 (5)	0 (0)	2 (1)
Continuous and time to event	0 (0)	0 (0)	1 (5)	0 (0)	1 (5)	0 (0)	2 (1)
Continuous and binary	0 (0)	0 (0)	0 (0)	0 (0)	0 (0)	1 (7)	1 (1)
Continuous, binary and time to event	1 (2)	0 (0)	0 (0)	0 (0)	0 (0)	1 (7)	2 (1)
Trial intervention							
Drug	59 (98)	10 (67)	19 (100)	7 (54)	19 (86)	9 (64)	123 (86)
Device	0 (0)	2 (13)	0 (0)	2 (15)	2 (9)	4 (29)	10 (7)
Diet	0 (0)	1 (7)	0 (0)	0 (0)	0 (0)	0 (0)	1 (1)
Psychological	0 (0)	0 (0)	0 (0)	1 (8)	0 (0)	0 (0)	1 (1)
Physiological	0 (0)	0 (0)	0 (0)	1 (8)	0 (0)	0 (0)	1 (1)

continued on next page

continued from previous page

Variable	Phase II		Phase II/III		Phase III		All Phases
	Private (n=60)	Public (n=15)	Private (n=19)	Public (n=13)	Private (n=22)	Public (n=14)	Total (n=143)
Drug and device	0 (0)	1 (7)	0 (0)	1 (8)	1 (5)	0 (0)	3 (2)
Education and diet	0 (0)	0 (0)	0 (0)	1 (8)	0 (0)	0 (0)	1 (1)
Drug and diet	1 (2)	1 (7)	0 (0)	0 (0)	0 (0)	1 (7)	3 (2)
Number of trial arms[4]							
1	16 (27)	8 (53)	5 (26)	9 (69)	13 (59)	11 (79)	62 (43)
2	14 (23)	4 (27)	7 (37)	2 (15)	6 (27)	1 (7)	34 (24)
3	10 (17)	3 (20)	4 (21)	0 (0)	2 (9)	0 (0)	19 (13)
4	9 (15)	0 (0)	2 (11)	1 (8)	1 (5)	1 (7)	14 (10)
≥ 5	11 (18)	0 (0)	1 (5)	1 (8)	0 (0)	1 (7)	14 (10)
Duration of primary outcome (days)	60 {76}	120 {315}	84 {88}	365 {29}	336 {214}	88 {252}	84 {224}
Sample size	200 {178}	138 {150}	306 {667}	300 {849}	451 {534}	1417 {2459}	240 {368}

Results presented as n (%) or median {IQR}.
[1] Other therapeutic areas include: detached placenta, gastric ulcers, venous thromboembolism, lower urinary tract symptoms, septic shock, visceral leishmaniasis, chronic abacterial prostatitis, anemia, primary visceral leishmaniasis, ulcer and pre-eclampsia.
[2] As of September 2014.
[3] > 50 only trials not included in ≥ 16 category.
[4] Excludes comparator arm. Number of arms in multi-arm multi-stage (MAMS) design taken to be the original starting number.
